# BSA Hydrogel Beads Functionalized with a Specific Aptamer Library for Capturing *Pseudomonas aeruginosa* in Serum and Blood

**DOI:** 10.3390/ijms222011118

**Published:** 2021-10-15

**Authors:** Markus Krämer, Ann-Kathrin Kissmann, Heinz Fabian Raber, Hu Xing, Patrizia Favella, Ingrid Müller, Barbara Spellerberg, Tanja Weil, Dennis Kubiczek, Susanne Sihler, Ulrich Ziener, Frank Rosenau

**Affiliations:** 1 Institute of Pharmaceutical Biotechnology, Ulm University, Albert-Einstein-Allee 11, 89081 Ulm, Germany; Markus-1.Kraemer@uni-ulm.de (M.K.); ann-kathrin.kissmann@uni-ulm.de (A.-K.K.); Heinz.Raber@uni-ulm.de (H.F.R.); dolphinzyt2418@gmail.com (H.X.); dennis.kubiczek@gmx.de (D.K.); 2 Department of Life Sciences, Albstadt-Sigmaringen University of Applied Sciences, 72488 Sigmaringen, Germany; Patrizia.Favella@uni-ulm.de (P.F.); mueller@hs-albsig.de (I.M.); 3 Institute for Medical Microbiology and Hygiene, University Hospital Ulm, 89081 Ulm, Germany; Barbara.Spellerberg@uniklinik-Ulm.de; 4 Department Synthesis of Macromolecules, Max-Planck-Institute of Polymer Science, 55128 Mainz, Germany; Weil@mpip-mainz.mpg.de; 5 Institute of Organic Chemistry III-Macromolecular Chemistry and Organic Materials, Ulm University, 89081 Ulm, Germany; Susanne.Sihler@uni-ulm.de (S.S.); Ulrich.Ziener@uni-ulm.de (U.Z.)

**Keywords:** aptamers, hydrogel beads, *Pseudomonas aeruginosa*, sepsis

## Abstract

Systemic blood stream infections are a major threat to human health and are dramatically increasing worldwide. *Pseudomonas aeruginosa* is a WHO-alerted multi-resistant pathogen of extreme importance as a cause of sepsis. Septicemia patients have significantly increased survival chances if sepsis is diagnosed in the early stages. Affinity materials can not only represent attractive tools for specific diagnostics of pathogens in the blood but can prospectively also serve as the technical foundation of therapeutic filtration devices. Based on the recently developed aptamers directed against *P. aeruginosa*, we here present aptamer-functionalized beads for specific binding of this pathogen in blood samples. These aptamer capture beads (ACBs) are manufactured by crosslinking bovine serum albumin (BSA) in an emulsion and subsequent functionalization with the amino-modified aptamers on the bead surface using the thiol- and amino-reactive bispecific crosslinker PEG_4_-SPDP. Specific and quantitative binding of *P. aeruginosa* as the dedicated target of the ACBs was demonstrated in serum and blood. These initial but promising results may open new routes for the development of ACBs as a platform technology for fast and reliable diagnosis of bloodstream infections and, in the long term, blood filtration techniques in the fight against sepsis.

## 1. Introduction

Infections with resistant pathogens represent one of the major health issues of concern in the 21st century, especially with nosocomial infections being among the most challenging tasks to handle for healthcare facilities. While plenty of literature exists on this problem and hundreds of research facilities worldwide are working on the topic, no country or institution can claim to be able to solve this problem or to present an ultimate solution in a reasonable time for the future. According to the World Health Organization (WHO), infections are the second highest cause for mortality worldwide [1]. Correspondingly, the European Center for Disease Control (ECDC) stated that more than four million patients are affected by nosocomial infections every single year in Europe alone, with immune-suppressed patients being especially life-threatened [1]. A general and severe burden is sepsis, which is estimated to have affected 49 million individuals and was related to approximately 11 million potentially avoidable deaths worldwide in 2017 [2]. The WHO recognized that mortality in these systemic bloodstream infections is often caused not only by suboptimal quality of care, inadequate health infrastructure, and poor infection prevention measures but also by late diagnosis [2]. It has been estimated that treatment of patients with septicemia accounts for an annual cost of approximately USD 14 billion [3]. In early 2017, carbapenem-resistant strains of the pathogenic Gram-negative bacterium *P. aeruginosa* were classified to be among the most threatening pathogens with the highest demand for novel antibiotics by the WHO [4], together with other prominent microbial pathogens such as methicillin-resistant *Staphylococcus aureus* (MRSA) or pathogenic yeasts such as species from the genus *Candida*. *Pseudomonas* spp. is the cause of sepsis in 20% of the cases diagnosed by blood cultures, qualifying as the most abundant Gram-negative genus associated with sepsis [5]. However, the high mortality from blood infections is partly due to the inability to rapidly detect and identify the causative pathogen and thus treat patients with appropriate antibiotics in the early stages of infection [6]. This can require at least one day or more for precise diagnosis, increasing the chances of patient mortality [7]. As a consequence, considerable effort has been invested into the development of rapid, sensitive, and specific assays to detect these pathogens. Amplification-based molecular diagnosis methods such as polymerase chain reaction (PCR) can reduce the assay time to hours. However, this methodology is often limited and not sensitive enough to detect the low concentrations of bacteria, thus needing additional steps such as initial enrichment [8]. Moreover, such methods may require inactivation or removal of inhibitors from the sample [9]. Separation by particles equipped with affinity entities towards the suspect pathogen is an alternative for the isolation of target cells directly from samples by eliminating the components that interfere with the PCR and related techniques [10]. Affinity particles can be coated with affinity molecules such as antibodies, peptides, or oligonucleotides to bind cell surfaces and have been shown to be efficient for the isolation of different organisms [11]. Since the introduction of single-stranded nucleic acids as specific binding molecules in the early 1990s [12,13], these so-called aptamers have developed into a meanwhile accepted class of affinity molecules for different purposes. Conceptually being used like antibodies or other biological binding molecules, but being selected completely in vitro and subsequently usually being produced by solid phase synthesis (phosphoramidite method) as single-stranded RNA or DNA molecules, aptamers can be regarded as a gift of biological chemistry to the sciences with an invaluable potential [14,15]. Aptamers have opened new routes in diverse fields such as molecular diagnostics, optical and electronics, and sensor technology as tools in molecular biology and therapeutics or as a novel class of pharmaceutical compounds in drug development. A typical SELEX (systematic evolution of ligands by exponential enrichment) process quickly reduces the sequence space (and thus complexity but also the utilizable diversity) available for target binding from ~10^14^ sequences to one or very few individual molecules with 20 to 100 nucleotides without the need for laboratory animals or even physiological conditions in the experiments [16]. Although it appears logical to aim at the isolation of one or a few single aptamers to specifically bind single proteins, exposing only a very limited variety of epitopes, this attempt appears virtually paradoxical for cells, exposing uncountable epitopes in at least hundreds of different surface structures, including membrane proteins and lipopolysaccharides. The recently introduced variation of the SELEX process is based on monitoring the successive selection rounds by fluorescence measurements of the developing libraries. This FLuCell-SELEX was directed against *P. aeruginosa* and generated polyclonal aptamer libraries which were better suited to reliably bind carbapenem-resistant *P. aeruginosa* than the best individual aptamers identified by deep sequencing of the library and bioinformatic analyses [17]. In order to combine the advantages of using polyclonal libraries and the technical advantages of solid phase synthesis of aptamers, a focused library is used here, consisting of the eight best aptamers isolated from the polyclonal library. These aptamers were used in this study with amino group modifications to be immobilized by covalent crosslinking on the surface of hydrogel beads as affinity molecules. The beads were manufactured in an emulsion and were composed of BSA, using 1-ethyl-3-(3-dimethylaminopropyl) carbodiimide-hydrochloride (EDC) as a crosslinker/activator in the simple stirring process, delivering spherical particles in the high micrometer dimension. BSA hydrogel was chosen as the matrix since similar materials have been shown to be fully biocompatible and suited for cell culture applications as constituents of drug delivery composites or in novel smart wound dressings [18,19]. Moreover, BSA as a natural component of blood circulating in the bloodstream is an ideal candidate for manufacturing surfaces free of interactions with blood cells and has been used as a coating for medical devices [20].

## 2. Results

### 2.1. Preparation of BSA Hydrogel Beads and Their Stability in PBS-EDTA Buffer

**Figure 1:**
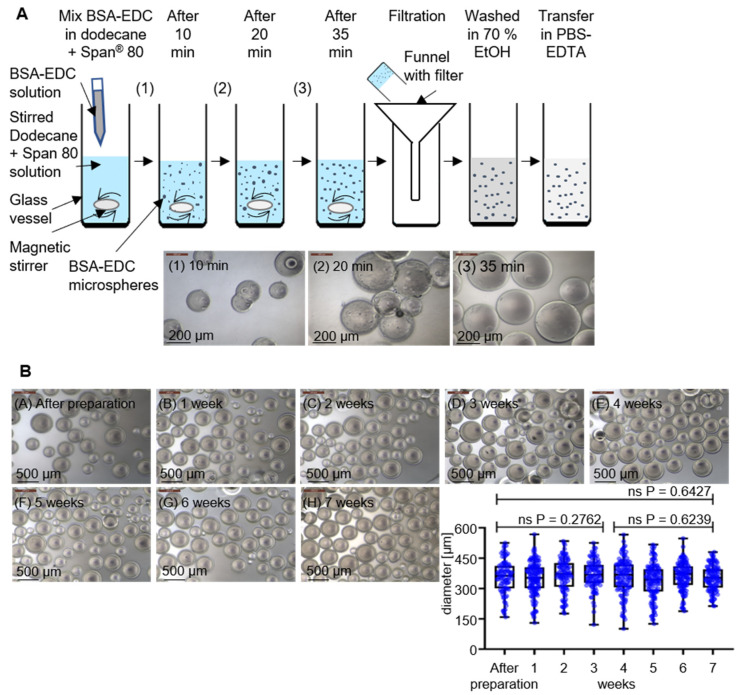
Preparation and stability analysis of BSA-EDC hydrogel beads. (**A**) Preparation of BSA-EDC beads through carboxy- and amino group crosslinking in dodecane and Span^®^ 80 with crosslinking stages of 10, 20, and 35 min and phase-contrast microscopy observation at 100× magnification. (**B**) Dimensional stability from BSA-based hydrogel beads over 7 weeks in PBS-EDTA buffer and phase-contrast microscopy visualization at 50× magnification for determination of the diameter stability. The determined *p*-values of unpaired t-tests with Welch’s correction show no significant (ns) changes in the diameter over the time.

The concept consists of two major technical components: novel BSA hydrogel-based beads and specific aptamer molecules as binding entities to functionalize a material with an affinity towards *P. aeruginosa* cells as a proof of concept for pathogen-capturing particles. The formation of micro hydrogel beads takes place via a crosslinking reaction of BSA molecules in a hydrophobic phase with the zero-length crosslinker 1-ethyl-3-(3-dimethylaminopropyl) carbodiimide-hydrochloride (EDC). EDC first reacts with a carboxyl group to form an amine-reactive O-acylisourea intermediate that quickly reacts with an amino group to form an amide bond and releases a non-toxic isourea byproduct which is removed by washing the hydrogel after fabrication [21,22]. A simple technology to produce spherical particles with potentially tunable sizes and excellent opportunities for subsequent upscaling of the procedure to industrially relevant scales is the use of emulsions. The hydrogel formation takes place in the dispersed aqueous phase, while the continuous oil phase comprises suitable surfactants as emulsifying agents with an impact on droplet sizes and the stability of the resulting emulsions. Stirring of a dodecane and Span^®^ 80 mixture (16.5 mL dodecane and 50 mg Span^®^ 80) at 1000 rpm on a magnetic stirrer resulted in the formation of an emulsion when the freshly mixed reaction solution of BSA (500 µL 20% (*w*/*v*) in aqueous MES buffer) and EDC (500 µL 10% (*w*/*v*) in aqueous MES buffer) was added quickly after mixing the protein solution and the crosslinker. The manufacturing procedure then allows harvesting of the hydrogel spheres by simple filtration (Figure 1A). The crosslinking reaction was analyzed for its completion by harvesting the reaction products after 10, 20, and 35 min and using microscopic inspection of the overall shape and determination of particle sizes using the FIJI image analysis software [23]. While the particles had average diameters of approximately 100 µm in the beginning, the size increased with stirring time to approx. 315 µm after 35 min. This final diameter was already reached after 20 min; however, at this time, considerable amounts of particles were irregularly shaped, which indicates imperfect separation of the beads in the emulsion. In addition, the appearance of the bead surfaces developed from rough to smooth between 20 and 35 min. Longer stirring times (>45 min), in contrast, resulted in visible damage of the particles and the beginning of structural disintegration (data not shown) (Figure 1A). The microscopic inspection of the surface appearance, shape, and particle integrity of particles washed in ethanol (70% (*v*/*v*)) and stored in PBS-EDTA buffer revealed a remarkable stability without significant changes for at least 7 weeks. This strongly suggests a promising application potential for these emulsion-derived particles as a raw material for subsequent functionalization reactions (Figure 1B).

### 2.2. Functionalization of BSA Beads and Their Specific Binding to P. aeruginosa in Different Constructs

**Figure 2:**
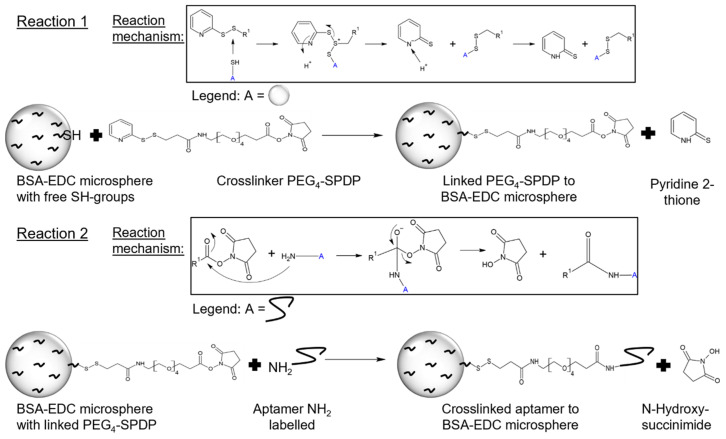
Functionalization of BSA hydrogel beads to aptamer capture beads (ACBs) with PEG_4_-SPDP- and NH_2_-labeled aptamer. The first reaction step is a displacement reaction of the sulfhydryl-reactive molecule (2-pyridyldithio group) of PEG_4_-SPDP with the free SH group of the BSA-EDC bead. In the second functionalization step, the NH_2_-labeled aptamer reacts with the amine-reactive portion (*N*-hydroxysuccinimide) of PEG_4_-SPDP via an NHS ester reaction [24].

Functionalization with the synthetic eight-aptamer library was achieved using the crosslinker PEG_4_-SPDP in a two-step reaction. PEG_4_-SPDP first binds to the thiol groups of BSA in a displacement reaction of the first sulfur atom, followed by the second reaction of the NHS ester being attacked by the amino group of the modified aptamers (Figure 2).

**Figure 3:**
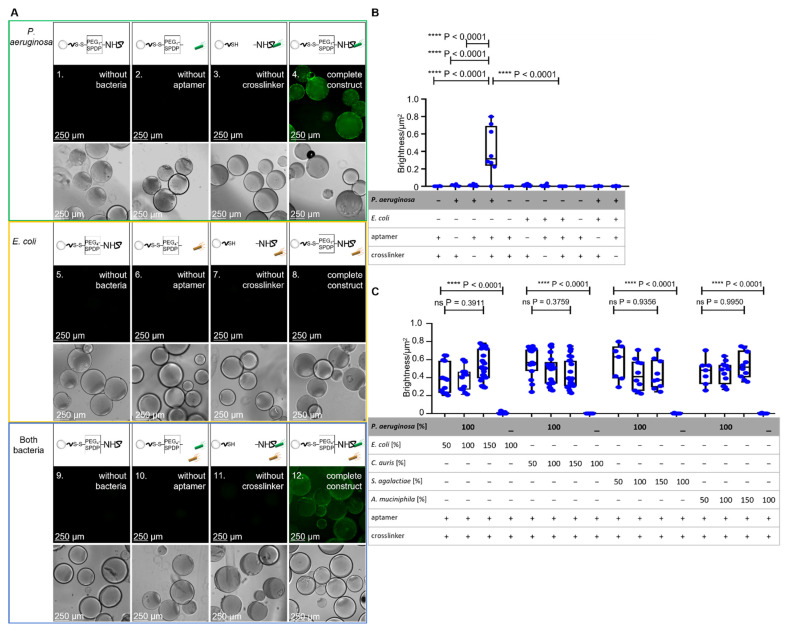
Analysis of incompletely and fully functionalized ACB constructs with PEG_4_-SPDP- and NH_2_-labeled aptamer. (**A**) Fluorescence microscopy and phase-contrast microscopy of the fully functionalized ACB constructs with GFP-modified *P. aeruginosa* and *E. coli* cells in comparison to incompletely functionalized constructs under fluorescence microscopy at 100× magnification. (**B**) BSF analysis of the different functionalized constructs with *P. aeruginosa* and *E. coli*. The statistical comparison of an unpaired t-test with Welch’s correction shows significant *p*-values of *p* < 0.0001 between the full constructs with *P. aeruginosa* and incompletely or fully functionalized constructs with *E. coli*. (**C**) BSF analysis of fully functionalized ACB constructs with different ratios of non-binding bacteria, including *E. coli*, *Candida auris*, *Streptococcus agalactiae*, and *Akkermansia muciniphila*. The statistical analysis with a Brown–Forsythe ANOVA shows no significant (ns) *p*-values over all *P. aeruginosa*-including compositions. The complete ACB constructs without *P. aeruginosa* show significant *p*-values of *p* < 0.0001.

Specific binding of *P. aeruginosa* cells (prototype strain PAO1) as the dedicated target of the aptamer-functionalized BSA beads was tested in a set of experiments based on fluorescence microscopy. A *P. aeruginosa* strain was used which harbors a plasmid for the expression of the green fluorescent protein (GFP) for labeling. While controls without the addition of the bacteria or as incomplete functionalization constructs without the addition of either aptamer or crosslinker (Figure 3(A1–A3)) showed no fluorescence, the addition of labeled bacteria resulted in a fluorescing halo around the beads, with individual cells being visible (Figure 3(A4)).

The halo was analyzed using the FIJI software such that the fluorescence intensity of the halo was quantified and correlated with the surface area of the beads (bead surface fluorescence analysis; BSFA). In contrast, when a recombinant probiotic *Escherichia coli* strain (Nissle 1917) also expressing GFP was used as a negative control in the same experimental setup, the fluorescent halo formation was not observed as expected, proving the intended specificity towards the dedicated specific target *P. aeruginosa*. When a mixture of *P. aeruginosa* and *E. coli* was used in a 1:1 ratio (100%:100%; 100% = 12,500 cells/mL), the halo was, as expected, not visibly reduced, and the bead fluorescence was also not reduced as expected as analyzed by FIJI-mediated intensity measurements with BSFA (Figure 3B,C).

The specificity experiments of Figure 3C were based on the *P. aeruginosa* target cells in mixtures with different ratios of non-binding bacteria. In order to determine the binding of *P. aeruginosa* to ACB, in this set of experiments an amount of GFP-labeled *P. aeruginosa* cells (12,500 cells/250 µL, (100%)) was used in the presence of the different cell counts of “contaminating” microbes (6250 cells/250 µL, (50%)), (12,500 cells/250 µL, (100%)) and (18,750 cells/250 µL, (150%)), although sepsis with multiple pathogens is truly not common. For the non-binding “contaminating” bacteria the pathogenic yeast *Candida auris,* the gram-positive *Streptococcus agalactiae* and the gram-negative human gut bacterium *Akkermansia muciniphila* as control pathogens were used. The fluorescence analyses with BSFA showed as expected, non-reduced fluorescent halos of the bound *P. aeruginosa* and no significant differences at all mixtures with *P. aeruginosa* and the control pathogens (all *p*-values > 0.37) (Figure 3C). Additionally, detectable fluorescence of the mixed experiment set ups is on the same level as the non-mixed experiment which only included the GFP modified *P. aeruginosa* The Fluorescence microscopy and phase-contrast microscopy pictures are shown in Figure S1.

### 2.3. Specificity of Fully Functionalized BSA Beads in Different Ratios of P. aeruginosa and Aptamers

**Figure 4:**
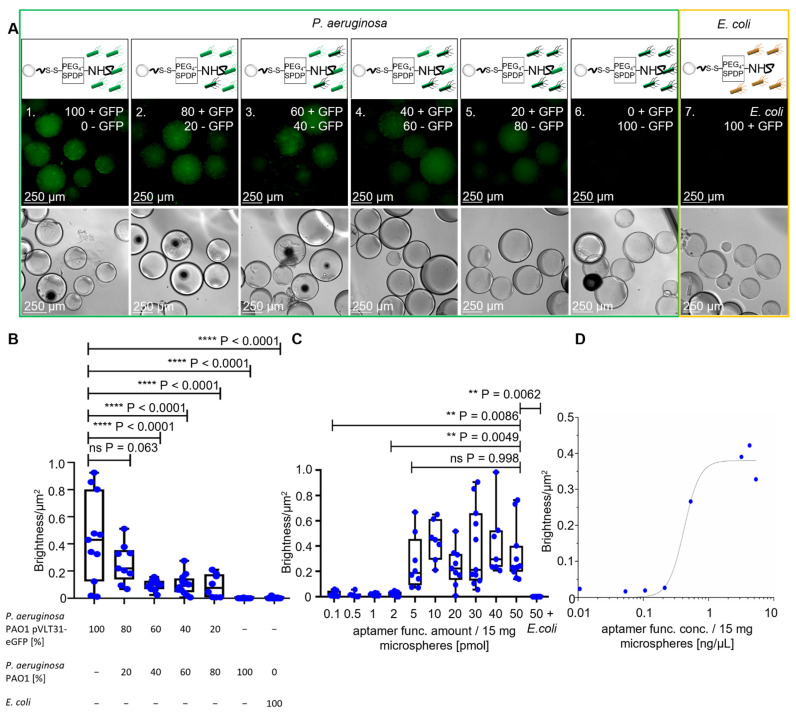
Specificity analysis with the full ACB constructs with different ratios of GFP-labeled *P. aeruginosa* cells and different amounts of aptamers. (**A**) Fluorescence microscopy and phase-contrast microscopy of the full ACB constructs with decreasing amounts of GFP-labeled *P. aeruginosa* PAO1 pVLT-31 eGFP and increasing unlabeled *P. aeruginosa* PAO1 cells, with a decreasing fluorescence halo at 100× magnification. (**B**) BSF analysis of the fluorescence microscopy images with different ratios of *P. aeruginosa.* The unpaired *t*-test shows *p*-values < 0.0001 between ratios of 100:60% or lower of GFP-labeled *P. aeruginosa* cells. (**C**,**D**) BSF analysis of the full ACB constructs with different functional aptamer amounts, with comparison through an unpaired *t*-test and a Hill plot to determine the binding capacity.

The fluorescent halos of ACBs with different ratios of GFP-labeled and unlabeled *P. aeruginosa* (Figure 4A), measured by BSFA, were statistically analyzed by an unpaired t-test with Welch’s correction and found to be significant with *p*-values < 0.0001 (Figure 4B), demonstrating a considerable sensitivity for quantitative analyses of mixed bacterial samples and the specificity of the *P. aeruginosa*-specific ACBs. The starting fabrication procedure used 50 pmol of amino-modified aptamers per functionalization reaction, also containing 15 mg of hydrogel beads with diameters of approximately 315 µm. Taking into account the Stokes radius of the globular BSA molecule of 3.48 nm [25], this amount of aptamers in the crosslinking reaction represents a 1:1 ratio of aptamers and BSA molecules accessible for the chemical modification on the surface of the beads. To prove the necessity of this high aptamer concentration for functionalization (i.e., the aptamer functionalization concentration = “apt func. conc”), independent batches of ACBs were prepared using different quantities of aptamers, ranging from 0.1 pmol up to the initial tentatively chosen 50 pmol per reaction (Figure 4C). BSF analyses of fluorescent halos (Figure S2) revealed that functionalization in the presence of 5 pmol aptamers delivered particles with a half-maximal binding capacity, whereas concentrations of 10 pmol and higher represented a plateau in the Hill plot (Figure 4D). This demonstrates that the amount of 50 pmol used in the standard functionalization reaction represents a considerable excess of aptamers, which is of importance in terms of resource efficiency and, in turn, also in terms of the economic feasibility of a potential future commercial product.

### 2.4. Binding of P. aeruginosa in Human Fluids such as Serum and Blood to Functionalized BSA Beads

**Figure 5:**
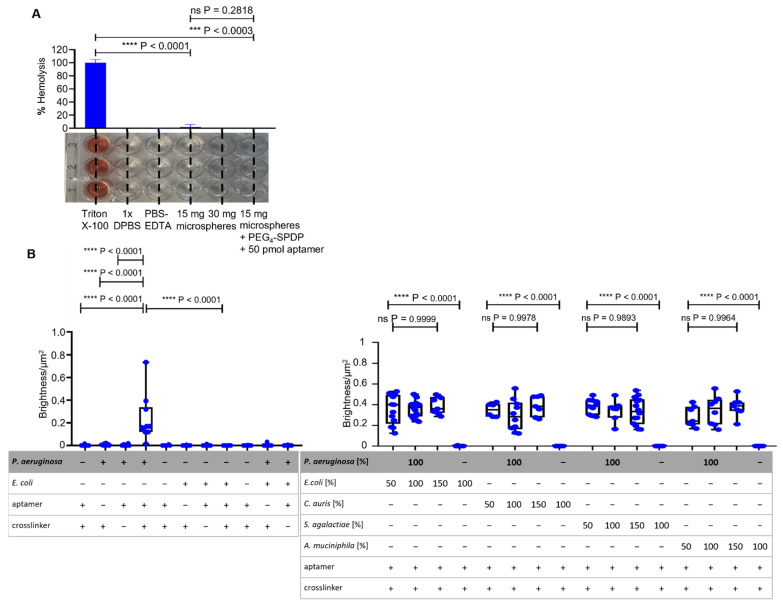

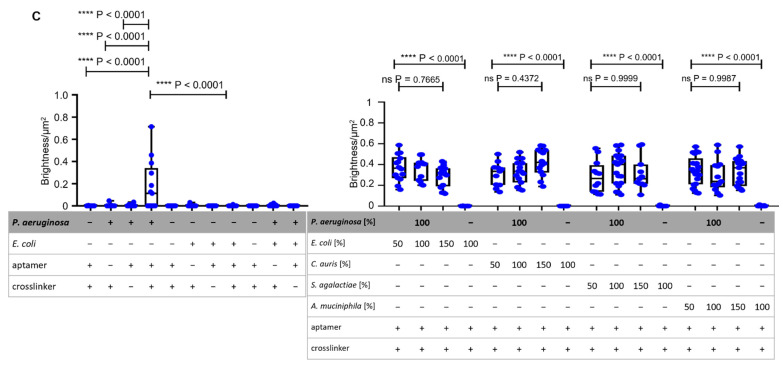
Compatibility of non-functionalized and fully functionalized BSA hydrogel beads and specific binding analysis with BSFA of ACBs in human serum and sheep blood. (**A**) Determination of sheep blood hemolysis with non-functionalized and fully functionalized hydrogel beads with absorption measurement at 450 nm. The detergent Triton^®^ X-100 acted as a positive reference, while DPBS acted as a negative reference [26]. (**B**) BSF analysis of ACBs in human serum with statistically analyzed unpaired t-tests with Welch’s correction between incomplete and fully functionalized ACBs with GFP-labeled *P. aeruginosa* and *E. coli* cells. For the setups of *P. aeruginosa* with different ratios of non-binding *E. coli*, *C. auris*, *S. agalactiae*, and *A. muciniphila*, the Brown–Forsythe ANOVA showed no significant *p*-values for all ratios (all *p* < 0.9893). (**C**) BSF analysis with statistically determined *p*-values via unpaired t-test with Welch’s correction of the specific binding of ACBs with *P. aeruginosa* and *E. coli* cells in sheep blood. Statistical analysis of the setups of different ratios of non-binding control strains mixed with *P. aeruginosa* was performed using a Brown–Forsythe ANOVA and shows no significant *p*-values (all *p* < 0.4372).

The intended application of the ACBs is for specific binding of *P. aeruginosa* cells in fluids of the (human) body. Thus, it is important that this binding can happen in serum and blood. In these experiments, human serum and sheep blood were the respective model body fluids. The stability of ACBs turned out to be not critical since semi-quantitative PCR analysis of the aptamers present on the beads showed that, after 8 or 16 h, the remaining concentrations were not reduced after incubation of ACBs in serum compared to control samples incubated identically in PBS-EDTA buffer (data not shown). This was encouraging enough to repeat the respective binding experiments and BSF analyses in serum and blood. Compared to full hemolysis upon addition of Triton^®^ X-100 as a surfactant positive control, neither the non-functionalized raw hydrogel beads nor the fully aptamer-functionalized and binding-competent ACBs caused detectable release of hemoglobin from erythrocytes (Figure 5A). Additional controls were the PBS-EDTA buffer used in the experiments presented here, which is also a part of the ACBs, and the commercial DPBS buffer used in the reference assay [26]. Both were found to be negative in the assay. As a major blood protein circulating in the blood stream and equipped by nature with non-binding properties towards cells, BSA has proven application potential as a coating material to improve the blood compatibility of (technical) surfaces [20,27,28,29]. Moreover, hydrogels manufactured from BSA by chemical crosslinking have proven to be biocompatible in cell cultures and other biotechnological applications [17,18,19,30,31,32].

The hemolysis results strongly indicate that the crosslinking methodology presented here based on EDC as a crosslinker as well as the use of the PEG_4_-SPDP bifunctional crosslinker in combination with the aptamers immobilized on the beads were highly compatible with blood. The binding capability of the ACBs towards *P. aeruginosa* cells was maintained with the same specificity as presented earlier in human blood serum, indicating that the native composition of this extracellular fluid including naturally occurring proteins, hormones, and other serum constituents is not critical for the specific molecular interactions of the immobilized aptamers and the *P. aeruginosa* epitopes on the cell surface (Figure 5B). However, the intensity (brightness) of the fluorescent halos upon binding of *P. aeruginosa* measured in serum (Figure S3A,B) was reduced to approximately 50% as compared to the set of experiments performed in PBS-EDTA buffer. Additionally, it can be determined that *P. aeruginosa* in the presence of different ratios of non-binding bacteria shows no different detectable fluorescent halos in comparison to the mixtures with *E. coli*, *C. auris*, *S. agalactiae*, and *A. muciniphila*. The same is true for the BSFA performed in blood (Figure S4), which showed a binding efficiency comparable to that observed in blood serum (Figure 5C). Correlating the fluorescence intensity of halos on the beads with the cell count on the bead surface suggests a calculated binding capacity of 25,000 *P. aeruginosa* cells per 15-milligram ACB in serum and blood which used in each of these experiments.

In patients with septicemia, the count of living bacterial cells varies significantly depending on the pathogen causing the sepsis, the stage of the infection, and the patient’s personal immunological fitness, with counts of colony-forming units (cfu) ranging from 1 to 1000 per milliliter of blood [33,34,35]. Although this system requires intensive follow-up characterization and optimization, the preliminary evidence presented here suggests that the binding capacity of the ACBs can allow the capturing of bacteria from a minimum of 25 mL of blood with short incubation times far beyond overnight cultivation. Standard procedures for sepsis diagnosis in clinical microbiology use blood cultures inoculated with large blood samples to provide enough bacteria to observe visible growth in reasonable incubation times, indicating that the bacterial count is normally extremely low and does not allow direct detection of the bacteria, e.g., by microscopy or even PCR techniques. Thus, enrichment prior to analysis can greatly increase the speed of sepsis diagnosis and thus the survival chances of patients. Techniques based on magnetic beads have been proposed which are based on particles coated with molecules that provide an affinity towards bacteria such as variants of lysozyme [36,37]. In contrast to these relatively unspecific binding molecules, aptamers can enable the development of species-specific capture particles, delivering a first line of evidence on the identity of the pathogen to increase the speed of the diagnostic workflow. The BSA hydrogel beads used here can be equipped with magnetic properties by including iron oxide particles in the emulsion during the crosslinking reaction, which can considerably increase their application potential. Although the hemolysis assay is far from being a comprehensive and final characterization of ACBs’ non-toxicity towards blood, the principal properties of BSA suggest a high degree of compatibility with human cells and thus probably with blood [17,18,19,20]. Biocompatible materials with a binding affinity towards pathogens, including bacteria, have entered the market and gained additional importance and recognition during the SARS-CoV-2 pandemic in blood filtration devices. These are based on heparin or polymyxin B and are thus relatively inert with human cells but can (unspecifically) interact with foreign cells or virus particles [38,39,40,41,42,43]. Although not demonstrated here, the use of aptamers on blood-compatible ACBs may enable the construction of filtration materials for efficient and also specific removal of pathogens from the blood of sepsis patients, thereby considerably extending the therapeutic repertoire in the fight against systemic infections.

## 3. Materials and Methods

### 3.1. Production of BSA-EDC Hydrogel Beads

To produce BSA-EDC hydrogel beads, first a 20% BSA (neoFroxx GmbH) and 10% EDC (Carl Roth GmbH + Co. KG) solution in MES buffer (100 mM) was prepared. Then, 50 mg of Span^®^ 80 (Carl Roth GmbH + Co. KG) was weighed into a glass vessel and 16.5 mL of dodecane (Sigma-Aldrich, Inc., Burlington, MA, USA) was added by pipette. Next, the dodecane–Span^®^ 80 mixture was mixed on a magnetic stirrer at 1000 rpm for 5 min. After that, 500 µL of the BSA solution and 500 µL of the EDC solution were briefly mixed in a 1.5-milliliter reaction tube and pipetted into the dodecane–Span^®^ 80 mixture while stirring at 1000 rpm. Then, the mixture was stirred for 35 min. After stirring, the beads were filtered by a pleated filter and washed 3 times in a new glass vessel with 10 mL of 70% EtOH. For preparation of the experiments, the BSA hydrogel beads were washed 3 times with 10 mL PBS-EDTA buffer (100 mM sodium phosphate, 150 mM NaCl, 1 mM EDTA, 0.02% sodium azide; pH 7.5).

#### Surface and Stability Analysis of BSA Hydrogel Beads

To investigate the polymerization stages, the polymerization reactions were stopped after 10, 20, and 35 min. After that, the beads were examined via phase-contrast microscopy at 100× magnification with the Leica DM IL inverted contrast microscope. For further determination of the stability of the BSA hydrogel beads in PBS-EDTA buffer, they were examined in intervals of 1 week for changes in the diameter or the surface or damage under the microscope at 50× magnification. The analysis of the diameter was performed with the image-processing software FIJI. For this, the scale of pixel/µm was set, and then the diameter of each hydrogel bead was measured. With GraphPad PRISM 8 and an unpaired t-test with Welch’s correction, the bead sizes were compared.

### 3.2. Specific Anti-P. aeruginosa PAO1 Aptamer PCR

To reproduce the eight specific aptamers against *P. aeruginosa* PAO1, a PCR was performed. The used forward primer was NH_2_-labeled ((5′[NH_2_]-TAG GGA AGA GAA GGA CAT ATG AT-3′), Eurofins Genomics Germany GmbH), while the reverse primer was labeled with biotin ((5′[Biotin]-TCA AGT GGT CAT GTA CTA GTC AA-3′), Eurofins Genomics Germany GmbH). The PCR was performed for each aptamer in 1× PCR buffer (1.5 mM MgCl_2_) containing 250 µM of each dNTP, 0.25 µM of each labeled primer, and 0.2 µM aptamer ssDNA. The used polymerase was Herculase II Fusion DNA polymerase (Agilent Technologies, Inc., Santa Clara, CA, USA). The amplification took place in the thermocycler SensoQuest Labcycler with an initial denaturation step at 94 °C for 3 min, followed by 25 cycles at 94 °C for 30 s for denaturation, 49.1 °C for 30 s for annealing, 72 °C for 30 s for elongation, and a final extension at 72 °C for 2 min. Following the PCR, the samples of the reaction tubes for each aptamer were pooled, and then gel electrophoresis was performed. For this, a gel with 2% agarose in 0.5% TBE buffer was prepared. Then, the gel was loaded with the samples consisting of 5 µL of dsDNA and 1 µL of 6X TriTrack DNA Loading Dye (Thermo Fisher Scientific, Inc., Waltham, MA, USA), and electrophoresis was carried out for 35 min at 150 V. In the next step, the gel was stained for 30 min in a 0.007% ethidium bromide bath. Afterwards, the gel was viewed and photographed under UV light (E-Gel^®^ Imager, Thermo Fisher Scientific, Inc.).

#### 3.2.1. Preparation of Aptamer ssDNA

To produce single-stranded DNA aptamers, in a new 1.5-milliliter reaction tube, first 50 µL of streptavidin-coated magnetic beads (BioMag^®^ Streptavidin, QIAGEN, Germantown, MD, USA) were washed 3 times with 1 mL 1× DPBS with the help of a magnetic separator. Then, the whole amplified aptamer dsDNA from Section 3.2 was added to the magnetic streptavidin beads and incubated while covered for 16 h at 22 °C on a rotator at 50 rpm. To remove unbound dsDNA, the supernatant was removed, and the magnetic streptavidin beads were washed with 1 mL 1× DPBS. In the following steps of strand separation, first 50 µL of NaOH (100 mM) was pipetted onto the magnetic streptavidin beads and incubated for 2 min without magnetic separation followed by a subsequent 2-min incubation with magnetic separation. After this, 45 µL of the NaOH solution was transferred into a new reaction tube with 126 µL of 1× DPBS, and 34.4 µL of NaH_2_PO_4_ buffer (100 mM) was added to the mix. The remaining 5 µL of ssDNA was used for the separation by gel electrophoresis as in Section 3.2. After the gel electrophoresis, the ssDNA concentration in ng/µL was measured using a Nanodrop photometer (Implen NP80).

#### 3.2.2. Functionalization of Aptamer ssDNA and BSA-EDC Hydrogel Beads

First, the prepared BSA-EDC hydrogel beads in PBS-EDTA buffer were filtered, and then 15 mg of beads were weighed into each needed well of a new 96-well plate. The beads were washed and resuspended 3 times in 250 µL PBS-EDTA buffer. In the next step, the supernatant was removed and 3.3 µL of PEG_4_-SPDP (Thermo Fisher Scientific) (20 mM in DMSO) was pipetted onto the beads, and the well was filled up to 250 µL with PBS-EDTA and incubated while covered at 22 °C for 16 h. After that, the beads were washed 3 times with 250 µL of PBS-EDTA buffer. Next, the eight aptamers were mixed in the desired concentration and then activated to ensure the right folding. For this, the aptamer mix was heated up to 95 °C for 5 min and then cooled for 5 min on ice. After that, the aptamer solution was covered and stored at 22 °C for 30 min. Then, the aptamer solution was added to the well and filled up to 250 µL with PBS-EDTA buffer and incubated while covered for 1 h at 22 °C. After incubation, the aptamer-crosslinked BSA-EDC hydrogel beads were washed 3 times with 250 µL PBS-EDTA buffer.

### 3.3. Triparental Mating of P. aeruginosa

To prepare a *P. aeruginosa* strain which is able to produce GFP, triparental mating was carried out. For this conjugation of 3 bacteria, *E. coli* Nissle pVLT31-eGFP was used as a donor strain, *P. aeruginosa* PAO1 was used as an acceptor strain, and *E. coli* DH5α pRK2013 was used as a carrier strain. First, one preculture of each strain was prepared by inoculating 5 mL LB medium (Carl Roth GmbH + Co. KG) with a single colony. After that, the optical density at 600 nm was measured with the VWR^®^ Spectrophotometer UV-1600PC, and 1 mL of bacteria suspension with OD_600_ = 0.6 was pipetted successively in one 1.5-milliliter reaction tube. For this, the bacterial suspension was centrifuged for 2 min at 11,000× *g* and the supernatant was removed. Then, the bacteria mix was resuspended in the last drop of the LB medium supernatant, pipetted on a prepared LB medium agar plate, and incubated for 3 h at 37 °C. After that, the bacteria colony was resuspended in 5 mL of LB medium with 50 µg/mL tetracycline (Carl Roth GmbH + Co. KG) and 100 µg/mL ampicillin (Carl Roth GmbH + Co. KG), then diluted to 10^5^–10^6^ to gain 10^3^ bacteria/mL and plated on agar plates with 50 µg/mL tetracycline and 100 µg/mL ampicillin. Additionally, an undiluted bacteria colony was plated on one new agar plate. Then, the plates were incubated at 37 °C for 16 h. After this, the grown *P. aeruginosa* PAO1 pVLT31-eGFP colonies were plated on a new agar plate with 50 µg/mL tetracycline and 100 µg/mL ampicillin to separate the colonies. After a renewed incubation for 16 h at 37 °C, these *P. aeruginosa* PAO1 pVLT31-eGFP bacteria colonies were used for the next experiments.

#### Bacteria Cultivation

For bacteria cultivation, a preculture for each bacterial strain was prepared. Therefore, 5 mL of LB medium was transferred into a test tube sterilized by autoclaving. For the bacteria *P. aeruginosa* PAO1 pVLT31-eGFP and *E. coli* Nissle pVLT31-eGFP, 10 µg/mL tetracycline was additionally added. To the control strain *P. aeruginosa* PAO1, no tetracycline was added. After adding the cells to the medium via inoculation loop, the precultures were incubated for 16 h at 37 °C with shaking at 150 rpm. In the next step, the OD_600_ values of the precultures were measured; then, 25 mL LB medium supplemented with 10 µg/mL tetracycline was inoculated to a starting OD_600_ of 0.05. No tetracycline was added to *P. aeruginosa* POA1 cultures without the plasmid pVLT31-eGFP. The cultivation was carried out at 37 °C with shaking at 150 rpm. After a cultivation time of 30 min and 1 to 7 h every hour, the OD_600_ was monitored. At OD_600_ = 0.6, the bacteria cultures of *P. aeruginosa* PAO1 pVLT31-eGFP and *E. coli* Nissle pVLT31-eGFP were induced with 0.4 mM Isopropyl-ß-d-1-thiogalactopyranoside (IPTG, Carl Roth GmbH + Co. KG). After induction, the cultivation was continued for another 3 h until the stationary phase of the bacteria was reached, and then the cells were counted under a microscope and a Thoma cell counting chamber. Then, the bacterial suspensions were diluted to 12,500 cells/250 µL for the following experiments.

### 3.4. Binding of P. aeruginosa PAO1 pVLT31-eGFP to Fully Functionalized ACBs

To determine the specific binding of *P. aeruginosa* PAO1 pVLT31-eGFP against the crosslinked aptamers to the BSA-EDC beads, various experiments were carried out. For this, the prepared beads from Section 3.2.2 and *P. aeruginosa* PAO1 pVLT31-eGFP bacteria from Section 3.3. were used. Additionally, *E. coli* Nissle pVLT31-eGFP, was used as control strain. The first control experiment had the fully functionalized ACB construct without *P. aeruginosa* or *E. coli* cells. For this, in two wells of a new 96-well plate, the BSA-EDC beads were prepared as described in Section 3.2.2. After washing 3 times with 250 µL PBS-EDTA, the wells were filled up to 250 µL with PBS-EDTA as a negative control and incubated while covered for 30 min at 22 °C. The second control experiment had an incomplete functionalization construct without NH_2_ aptamers but with *P. aeruginosa* or *E. coli* bacteria. Accordingly, in two wells, the BSA-EDC beads were crosslinked for 16 h with PEG4-SPDP as well but were not functionalized with NH_2_-labeled aptamers. After washing 3 times with 250 µL PBS-EDTA, 12,500 bacterial cells/250 µL of *P. aeruginosa* PAO1 pVLT31-eGFP, *E. coli* Nissle pVLT31-eGFP, or both were pipetted into the wells and incubated while covered for 30 min at 22 °C. The third control experiment had an incomplete ACB construct without the crosslinker PEG_4_-SPDP. For this functionalization, to the BSA-EDC beads, only 50 pmol of activated aptamer in 250 µL was added. After 1 h incubation time on BSA-EDC beads, the beads were washed 3 times with 250 µL PBS-EDTA. In the next step, 12,500 cells/250 µL of *P. aeruginosa* PAO1 pVLT31-eGFP, *E. coli* Nissle pVLT31-eGFP, or both were pipetted into the wells and incubated for 30 min while covered at 22 °C. The fourth experiment included a fully functionalized ACB construct and the bacteria cells. Therefore, the BSA hydrogel beads were functionalized as described above in Section 3.2.2. After washing 3 times with 250 µL PBS-EDTA, the wells were filled up with cells/250 µL (100%) of *P. aeruginosa* PAO1 pVLT31-eGFP, *E. coli* Nissle pVLT31-eGFP, *C. auris*, *S. agalactiae* BSU6, or *A muciniphila* mucT as control pathogens in ratios of 100:50, 100:100, and 100:150. Then, the samples were incubated while covered for 30 min at 22 °C. Afterwards, all samples were washed 3 times with 250 µL PBS-EDTA and then filled up to 250 µL with PBS-EDTA. In the next step, all samples were investigated by fluorescence microscopy and with phase-contrast microscopy at 100× magnification with the Leica DMi8 inverted fluorescent microscope. Then, the fluorescence microscopy images were analyzed using BSFA.

#### 3.4.1. Binding of *P. aeruginosa* PAO1 pVLT31-eGFP to Fully Functionalized ACBs in Comparison to Mixtures with Non-Targeted Bacteria

To further determine that other bacteria have no influence on the binding of *P. aeruginosa* to the ACBs, various unlabeled bacteria were used to produce bacterial mixtures with *P. aeruginosa* PAO1 pVLT31-eGFP. For this purpose, the hydrogel beads were first functionalized to ACBs as in Section 3.2.2. After functionalization, precultures of *P. aeruginosa* (LB medium), *C. auris* (YPD + 0.4% glucose medium), *S. agalactiae* BSU6 (TSB medium), and *A. muciniphila* mucT (5 mL Schaedler Bouillon + Resazurin (100 µg/mL) + 250 µL 1% mucin) were prepared. In the next step, the main cultures of the three aerobic bacteria were then prepared and proceeded as in Section 3.4. After expression, the bacteria cells were counted and different ratios of 6250 cells/250 µL (50%), 12,500 cells/250 µL (100%), and 18,750 cells/250 µL (150%) per bacterium were used so that the mixed samples of *P. aeruginosa* and the non-targeted bacteria had a maximal concentration of 31,250 cells/250 µL. In the next step, the samples were covered and incubated for 30 min at 22 °C, washed 3 times with 250 µL PBS-EDTA, and then filled up to 250 µL with PBS-EDTA. In the next step, all samples were examined with fluorescence microscopy and phase-contrast microscopy at 10× magnification, and all images were statistically analyzed by BSFA.

#### 3.4.2. Bead Surface Fluorescence Analysis (BSFA)

To statistically analyze the fluorescence halo of the bound bacteria, the image processing software FIJI was used. First, the scale of pixel/µm was set for determination of the visible area of the beads. In the next step, the color threshold was set, and the unselected dark areas were cut out. Then, the image was converted into a black and white saturation image via high saturation brightness Stack (HSB-Stack), and then the brightness/µm^2^ for each bead was measured. For the subsequent statistical evaluation, Prism 8 software and an unpaired *t*-test with Welch’s correction were used. For determination of the binding capacity, the number of fluorescent bacteria halos was analyzed. Therefore, the pixel/µm was set, and the fluorescence images were transformed into 16-bit black and white images. In the next step, the threshold was set, and the bacteria count/bead was measured using a particle-analyzing tool. The statistical analysis was carried out as mentioned above with Prism 8.

### 3.5. Binding Specificity Analysis of P. aeruginosa PAO1 pVLT31-eGFP and P. aeruginosa PAO1 in Different Ratios

For the following experiment, BSA-EDC hydrogel beads were prepared as described in Section 3.2.2 in the wells of a new 96-well plate. After full functionalization with PEG_4_-SPDP and 50 pmol NH_2_-labeled aptamer, the ACBs were washed 3 times with 250 µL PBS-EDTA. In the following step, 12,500 cells/250 µL of *P. aeruginosa* PAO1 pVLT31-eGFP and *P. aeruginosa* PAO1 were pipetted in different amounts to the fully aptamer-functionalized ACBs. The ratio of *P. aeruginosa* PAO1 pVLT31-eGFP decreased in 20% steps from 100 to 0%, while the amount of non-GFP-labeled *P. aeruginosa* PAO1 increased from 0 to 100%. The bacterium *E. coli* Nissle pVLT31-eGFP was used as a negative control. After covered incubation for 30 min at 22 °C, the ACBs were washed 3 times with 250 µL PBS-EDTA and then filled up to 250 µL with PBS-EDTA. In the next step, all samples were examined with fluorescence microscopy and phase-contrast microscopy at 10× magnification, and all images were statistically analyzed by BSFA.

### 3.6. Binding Specificity Analysis of P. aeruginosa PAO1 pVLT31-eGFP with Different Aptamer Concentrations

The binding capacity of the fully functionalized ACBs was investigated in the following experiment. BSA-EDC beads were prepared as described in Section 3.2.2 in the wells of a new 96-well plate. After the first functionalization with the crosslinker PEG_4_-SPDP and washing 3 times with 250 µL PBS-EDTA, the desired amounts of 0.1, 0.5, 1, 2, 5, 10, 20, 30, 40, and 50 pmol NH_2_-labeled aptamer were added to the beads for complete functionalization. Then, the samples were incubated while covered for 1 h at 22 °C. After incubation, the BSA hydrogel ACBs were washed 3 times with 250 µL PBS-EDTA. In the next step, cells/250 µL were pipetted to each sample and incubated for 30 min while covered at 22 °C. Then, the ACBs were washed 3 times with 250 µL PBS-EDTA and filled up to 250 µL with PBS-EDTA. In the next step, all ACB samples were examined with fluorescence and phase-contrast microscopy at 100× magnification. After that, the microscopy images were analyzed by BSFA.

### 3.7. Stability of Functionalized Aptamers on ACBs in Human Serum

To analyze the stability of the aptamers in human liquids such as human serum, first 15 mg of BSA beads were weighed into a 1.5-milliliter reaction tube and resuspended in 400 µL PBS-EDTA buffer. Then, the beads were washed 3 times with sterile PBS-EDTA buffer and functionalized with PEG_4_-SPDP and 50 pmol aptamer as described in Section 3.2.2. In the next step, the ACBs were washed 3 times with 400 μL sterile PBS-EDTA buffer and then resuspended in 400 μL human serum (Product Number H4522-20ML, Merck KGaA) or PBS-EDTA buffer as a control and incubated while covered at 22 °C for 0, 8, and 16 h. After this, the ACBs were washed 3 times with sterile Milli-Q water, and a PCR was carried out using the ACBs as a template in the reaction tubes. This PCR was performed in 1× PCR buffer (1.5 mM MgCl) containing 250 µM of each dNTP and 0.25 µM unlabeled forward primer (5′-TAG GGA AGA GAA GGA CAT ATG AT-3′) and reverse primer (5′-TCA AGT GGT CAT GTA CTA GTC AA-3′). The polymerase used was Herculase II Fusion DNA polymerase. The aptamer amplification took place in a SensoQuest Labcycler thermocycler with an initial denaturation step at 94 °C for 3 min, followed by 15 or 18 cycles at 94 °C for 30 s for denaturation, 49.1 °C for 30 s for annealing, and 72 °C for 30 s for elongation. After that, a final extension at 72 °C for 2 min was carried out.

### 3.8. Hemolysis Assay

First, 15 and 30 mg of non-functionalized beads were weighed into a 96-well (U-bottom) plate. Then, 15 mg of beads were weighed into the 96-well plate, which were then functionalized with PEG_4_-SPDP and 50 pmol aptamer as in Section 3.2.2. As a reference, 20% of the detergent Triton^®^ X-100 (positive reference), 1× DPBS (negative reference), and PBS-EDTA buffer were used. Here, 10 µL of each reference was pipetted into a 96-well plate. Then, 500 µL sheep blood (Product Number 10000100, Fiebig-Nährstofftechnik) was pipetted into two 1.5-milliliter reaction tubes. These were then centrifuged at 500× *g* and 4 °C for 5 min. The levels of plasma and hematocrit were marked, and the plasma supernatant was removed. After this, the blood was filled up to the marking of the plasma with 150 mM NaCl solution and carefully resuspended. Then, the tubes were centrifuged at 500× *g* and 4 °C for 5 min, and the washing step was repeated. After centrifuging again, the first reaction tube was filled up to the plasma marking with DPBS and the second with PBS-EDTA. Then, 250 µL from both sheep blood samples was diluted 1:50 in DPBS or PBS-EDTA. In the next step, 190 µL of the 1:50 diluted blood was transferred to the prepared samples. For the bead-including samples, the blood diluted in PBS-EDTA was used, while the DPBS-diluted blood was used for the references. Then, the samples were incubated for 1 h at 37 °C and centrifuged at 500× *g* for 5 min. The supernatants were removed and transferred into a new 96-well plate with a flat bottom, and the absorption at 450 nm was measured afterwards using a Tecan Spark microplate reader. After the measurement, the absorption was statistically analyzed with Prism 8 and an unpaired *t*-test with Welch’s correction.

### 3.9. Binding of P. aeruginosa PAO1 pVLT31-eGFP to BSA-EDC Hydrogel ACBs in Human Serum and Sheep Blood

To determine the specific binding of *P. aeruginosa* PAO1 pVLT31-eGFP to the fully functionalized ACB constructs in human serum or sheep blood, an experiment with incomplete and complete constructs was carried out as in Section 3.4 and with different ratios of the control pathogens as in Section 3.4.1 for the preparation of diluted bacteria samples. For the bacteria, suspensions of 6250 cells/250 µL (50%), 12,500 cells/250 µL (100%), and 18,750 cells/250 µL (150%) human serum or sheep blood were used instead of PBS-EDTA buffer.

## 4. Conclusions

Novel BSA beads coated with anti-*P. aeruginosa* aptamers for capturing cells of this pathogen in human fluids (aptamer capture beads; ACBs) were tested on the surface for functionality in human serum and sheep blood and were found to bind *P. aeruginosa* with high capacity and specificity. These initial results suggest that the ACBs exemplified here with aptamers against *P. aeruginosa* may open new avenues for the development of a platform technology that, when the repertoire is expanded by other aptamers or aptamer libraries evolved against pathogens of choice, may become of general importance. This technology promises not only fast and reliable enrichment of pathogens from human fluids to speed up diagnostic workflows, but also a flexibility which may meet the demands of fast assay development in clinical diagnostics for the beginning of the post-SARS-CoV-2 era to prepare healthcare systems for future pandemics with yet unknown pathogens.

## Data Availability

The data presented in this study are available in article and Supplementary Materials.

## Supplementary Materials

The following are available online at https://www.mdpi.com/article/10.3390/ijms222011118/s1.
