# Supplementary material for: BSA Hydrogel Beads Functionalized with a Specific Aptamer Library for Capturing Pseudomonas aeruginosa in Serum and Blood
Source: Int J Mol Sci. 2021 Oct 15;22(20):11118. doi: 10.3390/ijms222011118 (PMC8537436; doi:10.3390/ijms222011118)
Supplement: Supplementary file 1 [file ijms-22-11118-s001.zip › ijms-1301330-supplementary.pdf]

Supplementary Materials

P. aeruginosa + *E. coli*				
P. aeruginosa [%]	100	100	100	-
E. coli [%]	50	100	150	100

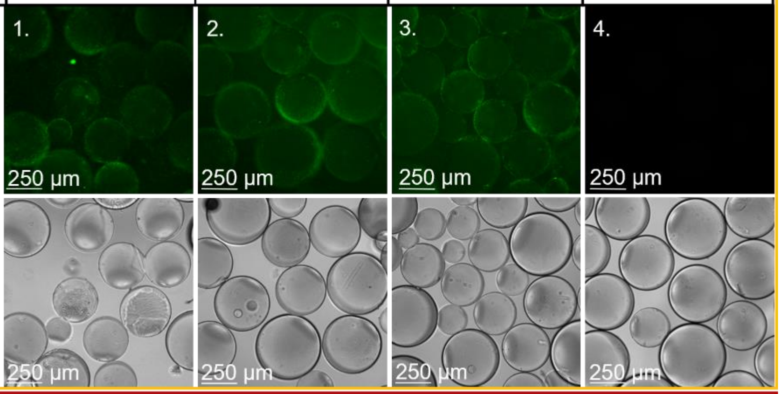

P. aeruginosa + *C. aruis*				
P. aeruginosa [%]	100	100	100	-
C. auris [%]	50	100	150	100

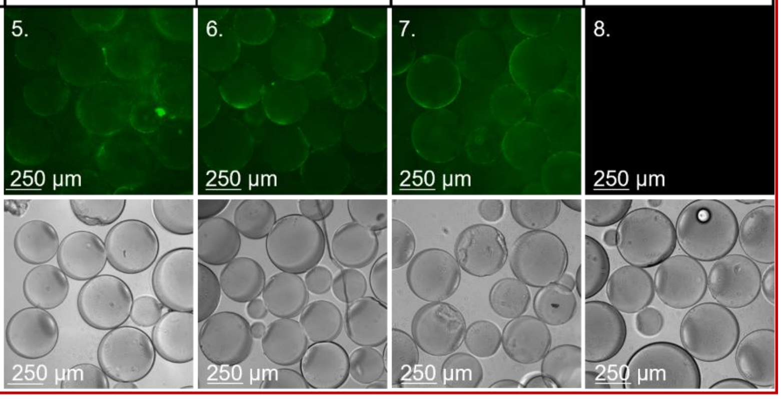

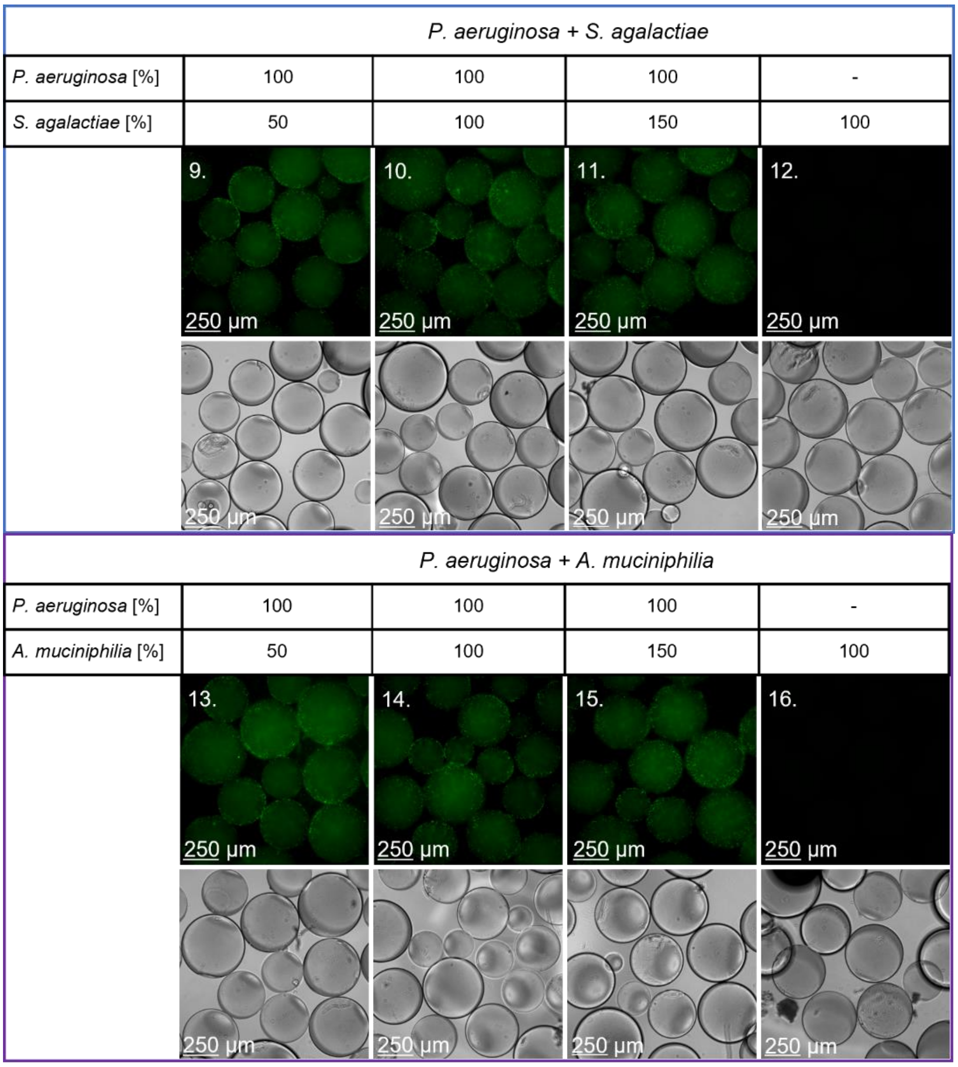

Figure S1. Fully functionalized ACB constructs with PEG_4-SPDP- and NH_2-labeled aptamer with *P. aeruginosa* in different ratios of various bacteria as the control strain. Phase-contrast and fluorescence microscopy at 100× magnification of the fully functionalized ACB constructs with GFP-modified *P. aeruginosa* and *E. coli*, *C. auris*, *S. agalactiae*, or *A. miciniphilia* cells in different ratios of the control pathogens for comparison of the binding efficiency.

All setups with GFP-modified *P. aeruginosa* and the control strains in different ratios showed no detectable differences in the visible halos of the bound *P. aeruginosa* bacteria (Figure S1(1–3, 5–7, 9–11, 13–15)). The experiments which only included one of the control strains and no GFP-modified *P. aeruginosa* showed no detectable fluorescent halos (Figure S1(4, 8, 12, 16)).

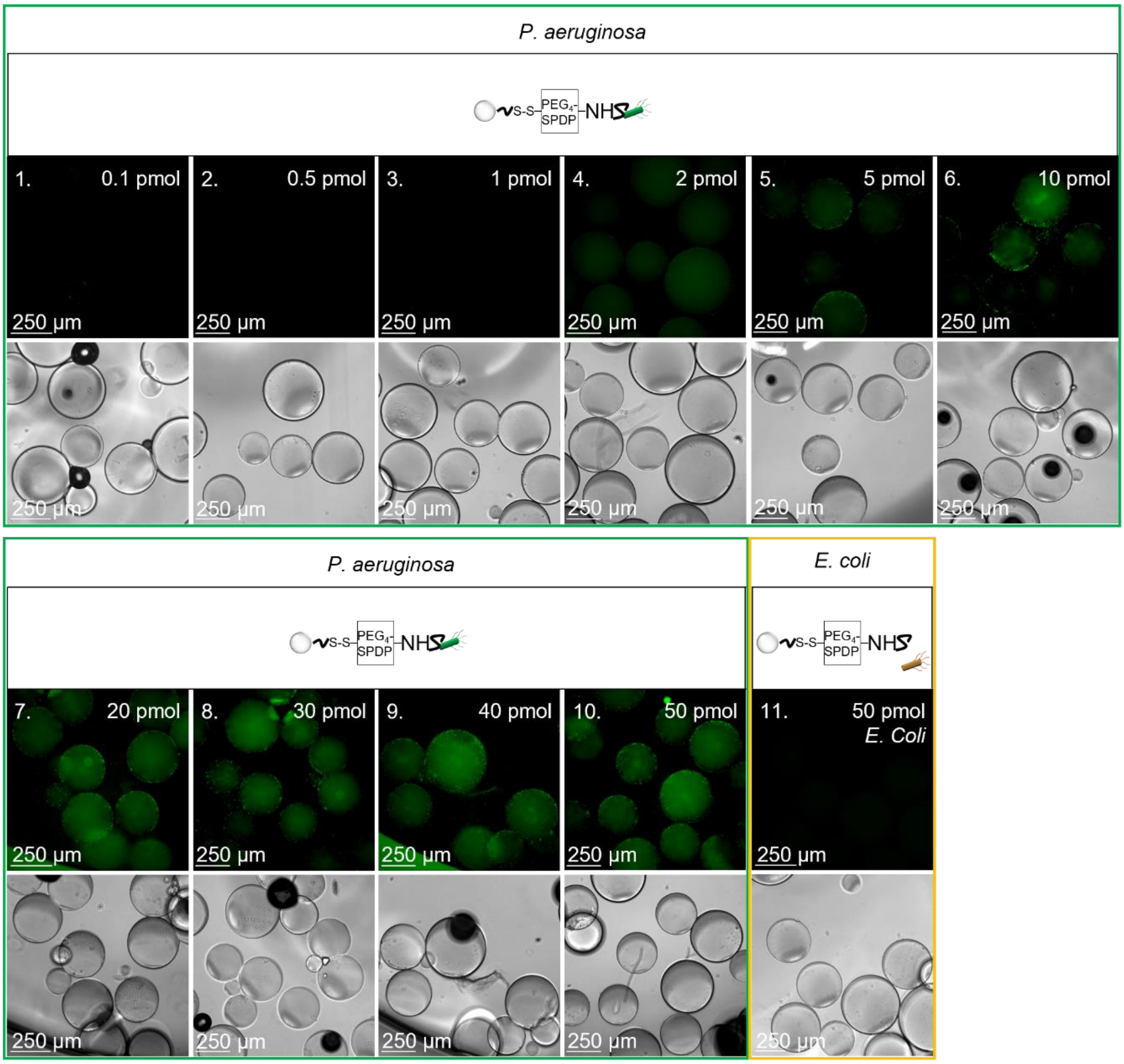

Figure S2: Fully functionalized ACB constructs with different amounts of aptamer. Fluorescence microscopy and phase-contrast microscopy at 100× magnification with GFP-labeled *P. aeruginosa* PAO1 pVLT-31 eGFP and, as a negative control, *E. coli Nissle* pVLT-31 eGFP cells of the fully functionalized ACB constructs with increasing amounts of NH_2-labeled aptamer which were used for functionalization.

The increasing amounts of aptamers showed an effect on the amount of binding of *P. aeruginosa* cells, where more cells were bound to the beads and a brighter halo of fluorescence could be detected until the plateau at 10 pmol. The negative control with *E. coli* and 50 pmol of specific aptamer showed no detectable fluorescence (Figure S2(11)).

A

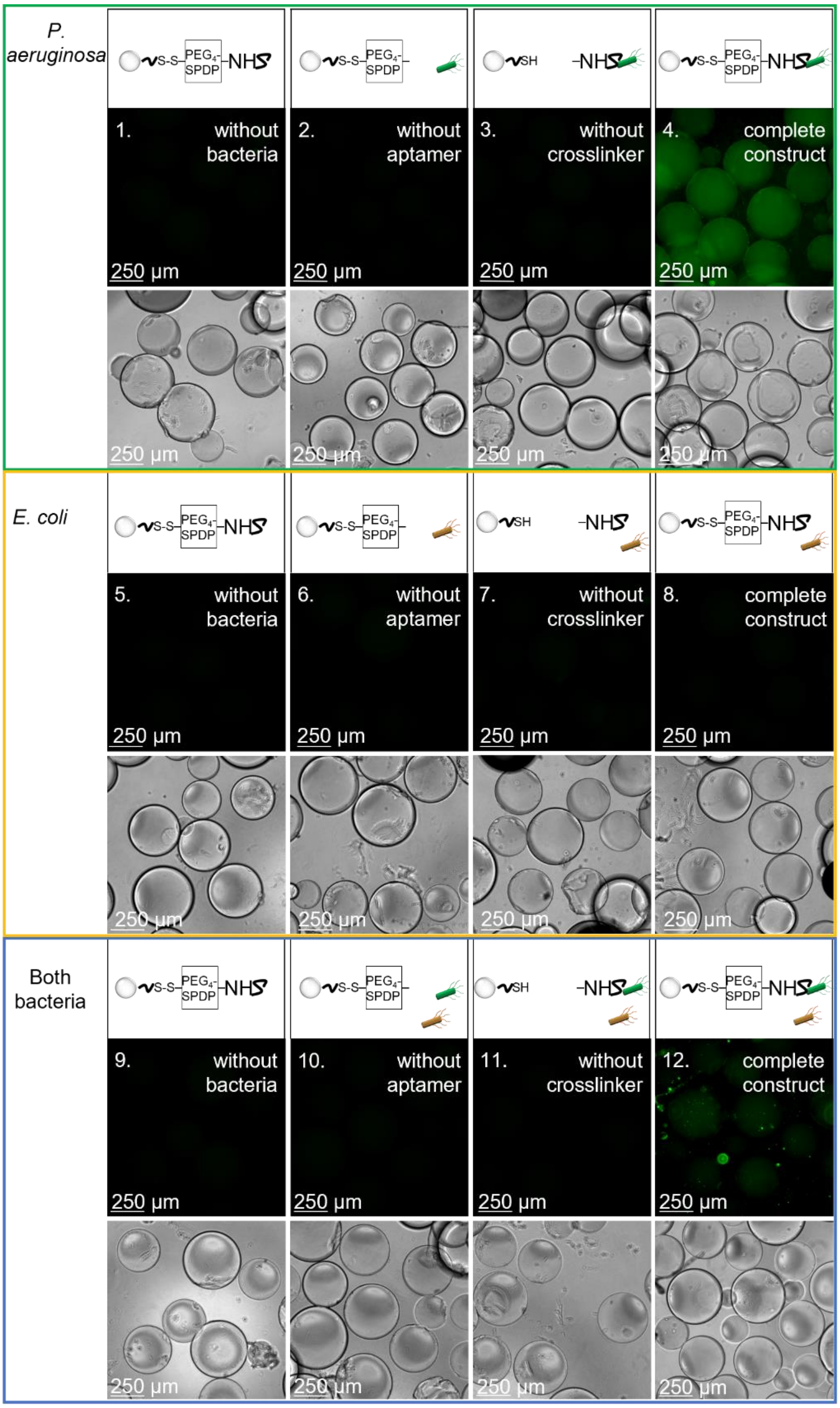
P. aeruginosa
E. coli
Both bacteria
1. without bacteria
2. without aptamer
3. without crosslinker
4. complete construct
5. without bacteria
6. without aptamer
7. without crosslinker
8. complete construct
9. without bacteria
10. without aptamer
11. without crosslinker
12. complete construct
S-S-
PEG4-SPDP
-NHS
SH
250 µm

B

P. aeruginosa + E. coli

P. aeruginosa [%]	100	100	100	-
E. coli [%]	50	100	150	100

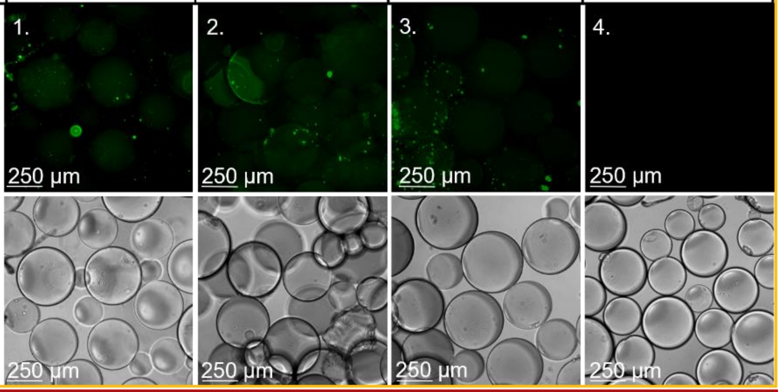

P. aeruginosa + C. aruis

P. aeruginosa [%]	100	100	100	-
C. auris [%]	50	100	150	100

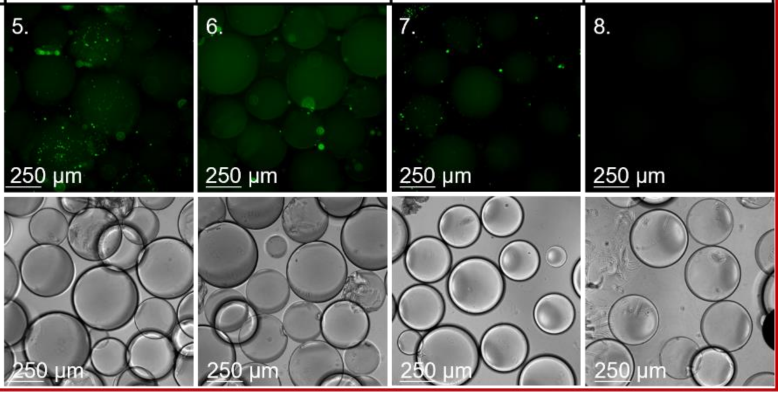

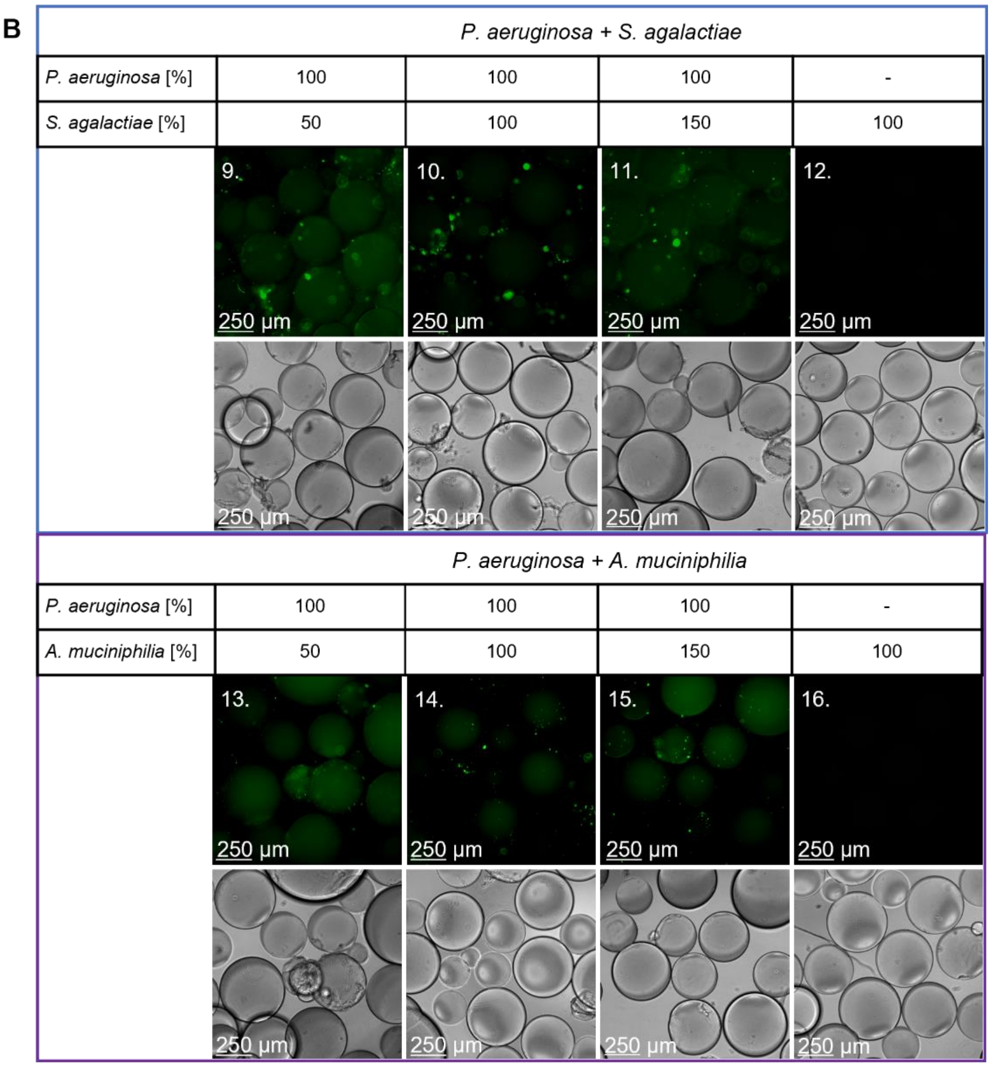

Figure S3. Incompletely and fully functionalized BSA-EDC bead constructs with PEG_4-SPDP- and NH_2-labeled aptamers in human serum. (**A**) Fluorescence microscopy of the fully functionalized ACB constructs with GFP-modified *P. aeruginosa* and *E. coli* cells in comparison to incompletely functionalized constructs under fluorescence microscopy at 100× magnification. (**B**) Fluorescence and phase-contrast microscopy of the fully functionalized ACB constructs with GFP-modified *P. aeruginosa* mixed with four control pathogens including *E. coli*, *C. auris*, *S. agalactiae*, and *A. muciniphila* in different ratios (50/100/150%).

In human serum, the different incomplete ACB constructs showed no binding of the *P. aeruginosa* cells, and no halo could be detected (Figure S3(A1–A3)), whereas the fully functionalized ACB constructs with the *P. aeruginosa* bacteria showed detectable fluorescent halos in unmixed and mixed setups with *E. coli* (Figure S3(A4, A12)). The negative control with fully functionalized ACB and *E. coli* showed no detectable halos (Figure A3(A5–A8)). The different setups of *P. aeruginosa* mixed with other control strains showed no visibly different halos of the bound bacteria (Figure S3(B1–B3, B5–B7, B9–B11, B13–B15)). The setups with only the control strains showed no detectable halos (Figure S3(B4, B8, B12, B16)).

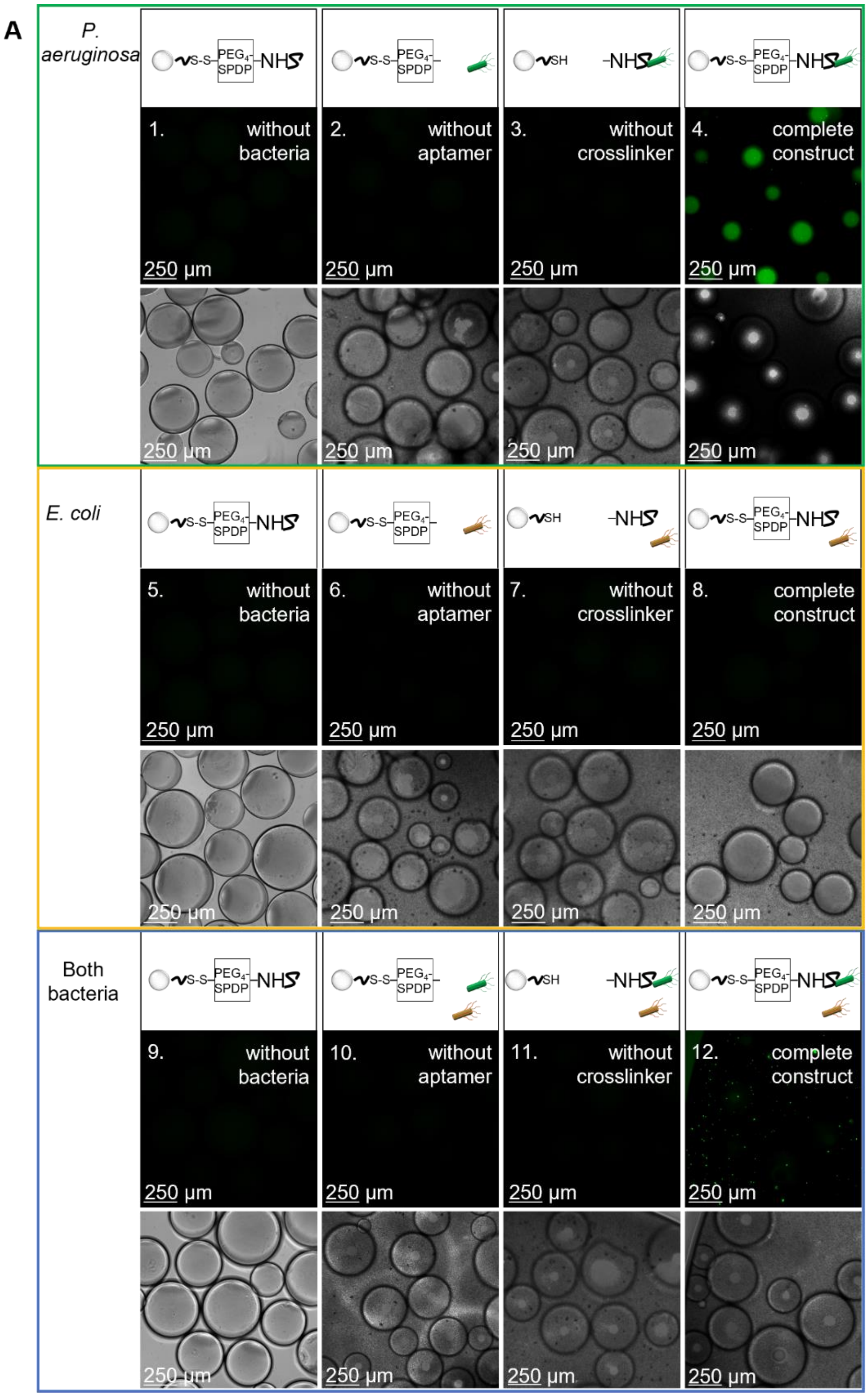

A
P. aeruginosa
E. coli
Both bacteria
PEG4-SPDP
NHS
SH
1. without bacteria
2. without aptamer
3. without crosslinker
4. complete construct
5. without bacteria
6. without aptamer
7. without crosslinker
8. complete construct
9. without bacteria
10. without aptamer
11. without crosslinker
12. complete construct
250 µm

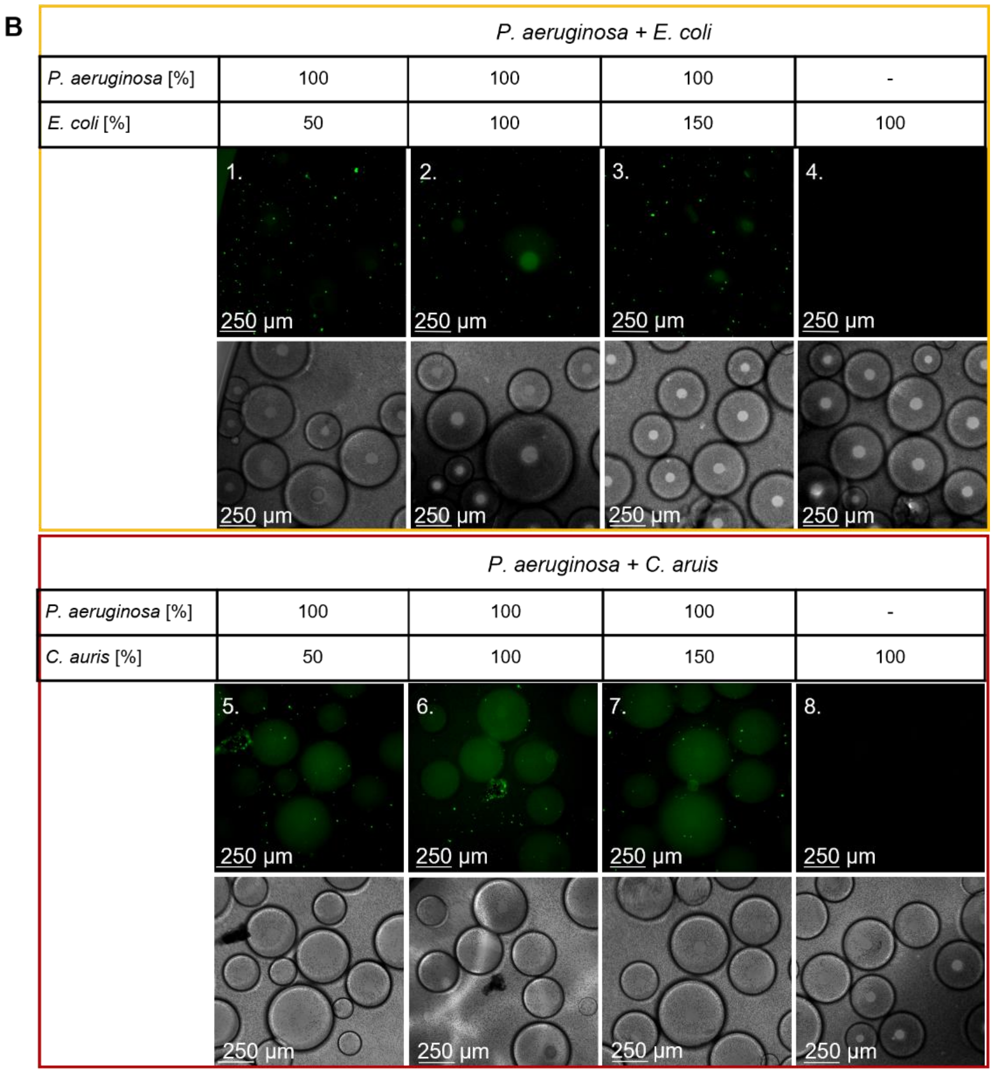
B
P. aeruginosa + E. coli
P. aeruginosa [%] 100 100 100 -
E. coli [%] 50 100 150 100
1.
2.
3.
4.
250 µm
P. aeruginosa + C. aruis
P. aeruginosa [%] 100 100 100 -
C. auris [%] 50 100 150 100
5.
6.
7.
8.
250 µm

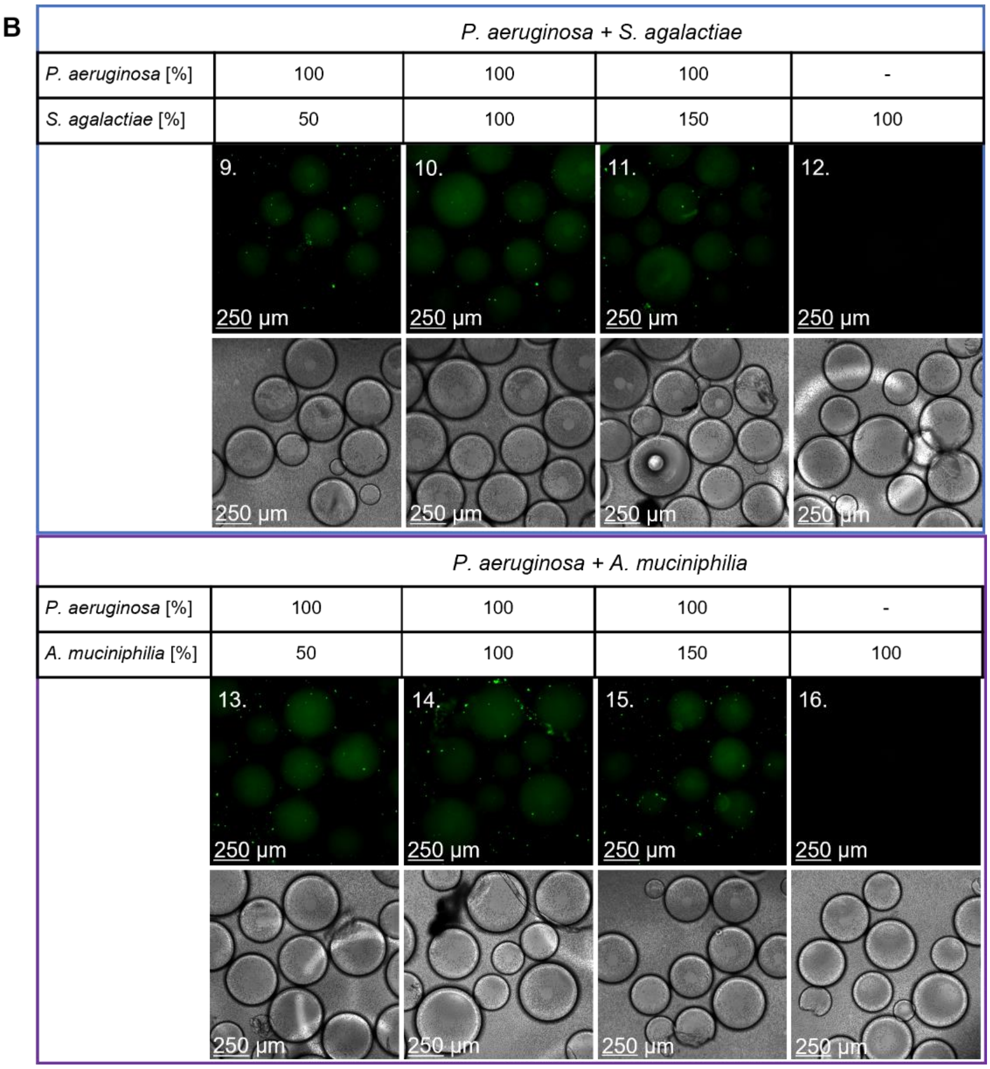

Figure S4. Incompletely and fully functionalized BSA-EDC bead constructs with PEG_4-SPDP- and NH_2-labeled aptamer in sheep blood. (**A**) Fluorescence microscopy of the fully functionalized ACB constructs with GFP-modified *P. aeruginosa* and *E. coli* cells in comparison to incomplete functionalized constructs under fluorescence microscopy at 100× magnification. (**B**) Fluorescence and phase-contrast microscopy of the fully functionalized ACB constructs with GFP-modified *P. aeruginosa* mixed with four control pathogens including *E. coli*, *C. auris*, *S. agalactiae*, and *A. muciniphila* in different ratios (50/100/150%).

The phase-contrast and fluorescence images of incompletely functionalized ACB constructs show no detectable fluorescent halos of *P. aeruginosa* (Figure S4(A1–A3)), whereas the unmixed and mixed fully functionalized ACBs with *P. aeruginosa* cells show detectable fluorescent halos (Figure S4(A4, A12)). The negative control with *E. coli* cells and incomplete and complete ACBs shows no detectable fluorescent halos (Figure S4(A5–A8)). The setups of *P. aeruginosa* mixed with different control strains in different ratios show various halos of the bound bacteria (Figure S4(B1–B3, B5–B7, B9–B11, B13–B15)). The setups without *P. aeruginosa* and only with the control pathogenic strains show detectable halos of bound bacteria (Figure S4(B4, B8, B12, B16)).
